# The influence of affective state on exogenous attention to emotional distractors: behavioral and electrophysiological correlates

**DOI:** 10.1038/s41598-017-07249-x

**Published:** 2017-08-14

**Authors:** Alejandra Carboni, Dominique Kessel, Almudena Capilla, Luis Carretié

**Affiliations:** 1 Universidad de la República del Uruguay, Montevideo, Uruguay; 2 Universidad Autónoma de Madrid, Madrid, Spain

## Abstract

The interplay between exogenous attention to emotional distractors and the baseline affective state has not been well established yet. The present study aimed to explore this issue through behavioral measures and event-related potentials (ERPs). Participants (N = 30) completed a digit categorization task depicted over negative, positive or neutral distractor background pictures, while they experienced negative, positive and neutral affective states elicited by movie scenes. Behavioral results showed higher error rates and longer reaction times for negative distractors than for neutral and positive ones, irrespective of the current emotional state. Neural indices showed that the participants’ affective state modulated N1 amplitudes, irrespective of distractor type, while the emotional charge of distractors modulated N2, irrespective of the emotional state. Importantly, an interaction of state and distractor type was observed in LPP. These results demonstrate that exogenous attention to emotional distractors is independent from modulating effects of the emotional baseline state at early, automatic stages of processing. However, attention to emotional distractors and affective state interact at later latencies.

## Introduction

Attention is preferentially directed to emotional stimuli, alerting organisms to important events in the environment (e.g., refs 1, 2). This rapid selection and evaluation of visual information facilitates adaptive reactions to novel and biologically relevant stimuli (such as danger, food or sexual stimuli). Stimuli capturing attention trigger automatic, stimulus-driven mechanisms, defined as exogenous attention, which can be conceptualized as a sort of interruption of endogenous attention to the ongoing task (by definition, controlled and goal driven) (e.g., refs 3, 4).

Several studies have reported behavioral and electrophysiological effects of enhanced attention to distractors with affective content. These effects have mainly been analyzed through concurrent, but distinct, target distractor (CDTD) paradigms (also named directed attention tasks^5^). In these tasks, targets (i.e., elements on the screen asked to be endogenously attended by experimental instructions; e.g., centrally-presented digits) and distractors (i.e., other visual elements, which are irrelevant to the task but which may potentially capture attention in an exogenous way; e.g., background pictures) appear at the same time but are physically segregated (please note that, given the simultaneous presentation of targets and distractors, CDTD tasks do not aim at differentiating between engagement and disengagement processes related to the distractor). At the behavioral level, automatic attention to emotional distractors is typically reflected in increased disruption in the ongoing task, typically observed in longer reaction times and higher error rates. Most existing studies points to a negativity bias: Negative distractors seem to interfere with performance in the main task to a greater extent than neutral or even positive ones. Thus, in studies where negative distractors were presented on the screen, reaction times and/or error rates in the ongoing task were reported as being significantly augmented compared to neutral and, in some studies, positive distractors^6–15^. In turn, studies showing largest effects due to positive^16^, or both negative and positive distractors^17–22^ are less numerous. Moreover, at the neural level, attentional biases towards emotional distractors have also been detected. Studies exploring temporal dynamics have usually reported a modulation at early stages of processing within the first 300 ms from stimulus onset. The main components of the event-related potential, which have been reported to be sensitive to attentional modulations by emotional distractors, are posterior P1, anterior P2, and the family of N2 components. Earliest evidence has been found in the P1 component, which usually peaks around 100 ms; specifically, larger P1 amplitudes at posterior topographies have been reported when negative, compared to neutral distractors were presented^23, 24^. Anterior P2 has shown largest amplitudes elicited by negative^9, 10, 20, 25^, positive^26^, or by both negative and positive distractors, compared to neutral ones^23^. Likewise, N2 ‒at wider topographies‒ has been reported to be sensitive to negative^6, 27, 28^, positive^23, 26^, or negative and positive distractors^29^ as well. The scarce data existing on late positivities in studies exploring attention to emotional distractors are focused on LPP. Specifically, one study provided evidence of enhanced posterior LPP amplitudes elicited by negative distractor pictures presented in the background^17^. Moreover, there are several other studies also reporting effects of emotional (negative and/or positive) distractors on LPP^26, 30–34^. In all these studies, emotional distraction was presented at fixation. Thus, voluntarily directed endogenous attention is most probably contributing to the results of these studies. In fact, these results are similar to the evidence obtained when stimuli were endogenously attended from the onset of the trial (i.e., largest LPP amplitudes in response to negative and positive stimuli; e.g., refs 35–42). The disrupting effects produced by emotional distractors reviewed above depend on several factors, including properties of the targets and distractors themselves, but also on the individual’s state and trait characteristics (see a review in ref. 43). In this context, the interaction of the emotional content of distractors with the individual’s affective state is still a very scarcely explored issue.

When emotional stimuli are presented as endogenously attended targets, there is growing evidence showing such an interaction between attentional processes and mood states. Generally, negative mood states have been found to impair attentional processes towards external stimuli, while positive states have been proposed to broaden attention (e.g., refs 44–46). When dealing with emotional information, behavioral data have revealed state-congruent biases; on the one hand, a positive state may lead to preferentially attend to positive targets^47, 48^; on the other hand, a negative state may intensify the vigilance to negative stimuli, while reducing the endogenous processing of positive ones^47, 49, 50^. However, there are other data showing that emotional states may improve attention to target stimuli of the opposite valence^51^. At the neural level, enhancing effects evoked by negative and positive mood on attention to emotional targets have been described at both early and late processing stages (P1^52^; LPP^50^). For emotional stimuli presented as distractors, which, by definition, are attended in an exogenous manner, previous evidence is remarkable scarce. Behavioral data suggest that these distractors may interact with the affective state^53^ and with the anxiety level of the individual^8^, eliciting both congruent and incongruent effects. At the neural level, the influence of affective state on attention allocation during target-distractor tasks, up to now, has only been explored for emotionally neutral task content^54–57^. These studies reported that mood may alter the earliest cortical stage of distractor processing, specifically, the centro-parieto-occipital C1 component. Moreover, previous behavioral and fMRI data demonstrate that affect may influence neural control processes underlying the resolution of cognitive interference^58^; thus, it has been observed that negative mood may disturb the selection of task-relevant information when presented with task-irrelevant emotional distraction.

However, to the best of our knowledge, no ERP studies have analyzed the particular neural time course of the interaction of mood and attention towards emotional distractors in healthy individuals while experiencing different affective states. Given the evidence described for endogenously attended emotional targets and the scarce results for emotionally neutral distractors, it may be hypothesized that mood will influence attention towards emotional distractors. Hence, the present study aimed to explore the behavioral and electrophysiological dynamics of the interaction between the basal affective state and attention to emotional distractors, combining both negative and positive valence. To this end, participants’ affective state was modulated by showing them short pleasant and unpleasant movie fragments. This kind of mood induction has been reported to be most effective in previous studies^59–61^. Affective state modulations were electrophysiologically controlled through peripheral indices: Skin conductance level; SCL. SCL has been employed to assess induced mood states in many previous studies (e.g., refs 62–64), and has been observed to be sensitive to participants’ arousal level^62, 65, 66^. Then, a concurrent but distinct target–distractor task was presented^43^, in which the participants’ endogenous attention was engaged in categorizing digits, while emotional and neutral background distractor pictures were concurrently presented. Behavioral (reaction times and error rates) and electrophysiological (ERP) indices were recorded. Based on previous evidence, it was hypothesized that emotional, compared to neutral distraction, would result in increased attention modulation observed in 1) longer reaction times and/ or higher error rates in the digit categorization task when emotional distractors were presented, and in 2) greater amplitudes of posterior P1, anterior P2, anterior/ posterior N2, and/ or posterior LPP. It was also expected that emotional movie clips would induce affective states, as indexed by 1) increased SCL, and 2) amplitude modulations of posterior C1. Furthermore, emotional states were hypothesized to interact with the emotional content of distractors, although no particular hypothesis on the direction of these effects could be formulated, given that previous evidence is contradictory, showing both state-congruent end state-incongruent effects.

## Results

### Behavioral data

Behavioral results are summarized in Table 1 (although statistical analyses were performed on normally transformed data ‒see Data Analysis section‒, this table shows original data for facilitating interpretation). Two-way repeated ANOVAs were computed on transformed data, introducing State (negative, neutral, positive) and Distractor (negative, neutral, positive) as factors. Results showed a significant main effect of Distractor on both RTs and ERs [*F*(2,58) = 8.9, *p* < 0.001, *η* ^*2*^_*p*_ = 0.234 and *F*(2,58) = 7.2, *p* < 0.01, *η* ^*2*^_*p*_ = 0.199, respectively]. Bonferroni-corrected post hoc tests indicated that significantly longer RTs and higher ERs were associated with negative distractors, compared to neutral and positive ones [all *p* < 0.05]. There were no significant differences for State, nor for the interaction of State and Distractor [all p > 0.05].

**Table 1:** Means and standard deviations (in parenthesis) of: (i) average number of trials, (ii) behavioral responses, and (iii) neural responses (factor scores, linearly related to amplitudes).

	Negative distracter						Neutral distracter						Positive distracter					
	Negative state		Neutral state		Positive state		Negative state		Neutral state		Positive state		Negative state		**Neutral state**		**Positive state**	
**Trials**																		
Average number of trials	31.7	(4.3)	32.9	(5.0)	31.9	(5.0)	33.4	(4.5)	33.0	(4.6)	33.3	(4.0)	33.3	(4.5)	32.9	(4.6)	33.2	(4.0)
Behavior*																		
Reaction times (ms)	1077	(228)	1106	(237)	1073	(213)	1067	(230)	1088	(237)	1059	(204)	1061	(226)	1076	(227)	1050	(202)
Error rates (%)	11.17	(7.33)	9.29	(7.17)	9.84	(5.29)	7.92	(7.55)	8.02	(6.71)	8.93	(6.68)	8.33	(6.90)	8.97	(8.11)	7.92	(6.06)
**Scalp level ERPs**																		
anterior N1 (factor scores)	−0.133	(1.168)	0.343	(0.881)	−0.033	(1.030)	−0.391	(0.998)	0.257	(1.024)	−0.183	(1.006)	−0.191	(1.019)	0.377	(0.906)	−0.047	(0.754)
posterior N1 (factor scores)	−0.072	(0.987)	0.166	(1.082)	−0.099	(1.146)	−0.212	(0.689)	0.193	(1.106)	−0.184	(1.005)	−0.004	(0.900)	0.277	(1.050)	−0.065	(0.975)
anterior N2 (factor scores)	0.050	(1.145)	0.041	(0.837)	−0.098	(0.916)	0.323	(1.262)	0.134	(0.832)	−0.085	(0.814)	0.144	(0.894)	−0.236	(1.039)	−0.272	(1.138)
posterior N2 (factor scores)	−0.026	(0.979)	-0.070	(0.849)	−0.216	(0.989)	0.335	(1.219)	0.188	(0.903)	0.018	(1.012)	0.122	(0.950)	−0.110	(0.993)	−0.242	(1.053)
posterior LPP (factor scores)	0.123	(1.026)	0.162	(0.998)	0.204	(0.965)	0.002	(0.942)	−0.302	(0.841)	−0.501	(0.989)	0.425	(1.001)	−0.094	(1.099)	−0.019	(0.936)

*To achieve normality, analyses were performed on transformed data: log10[RT]), arcsin[√ER].

### ERP data

#### Detection, spatio-temporal characterization, and quantification of relevant ERP components

**Figure 1:**
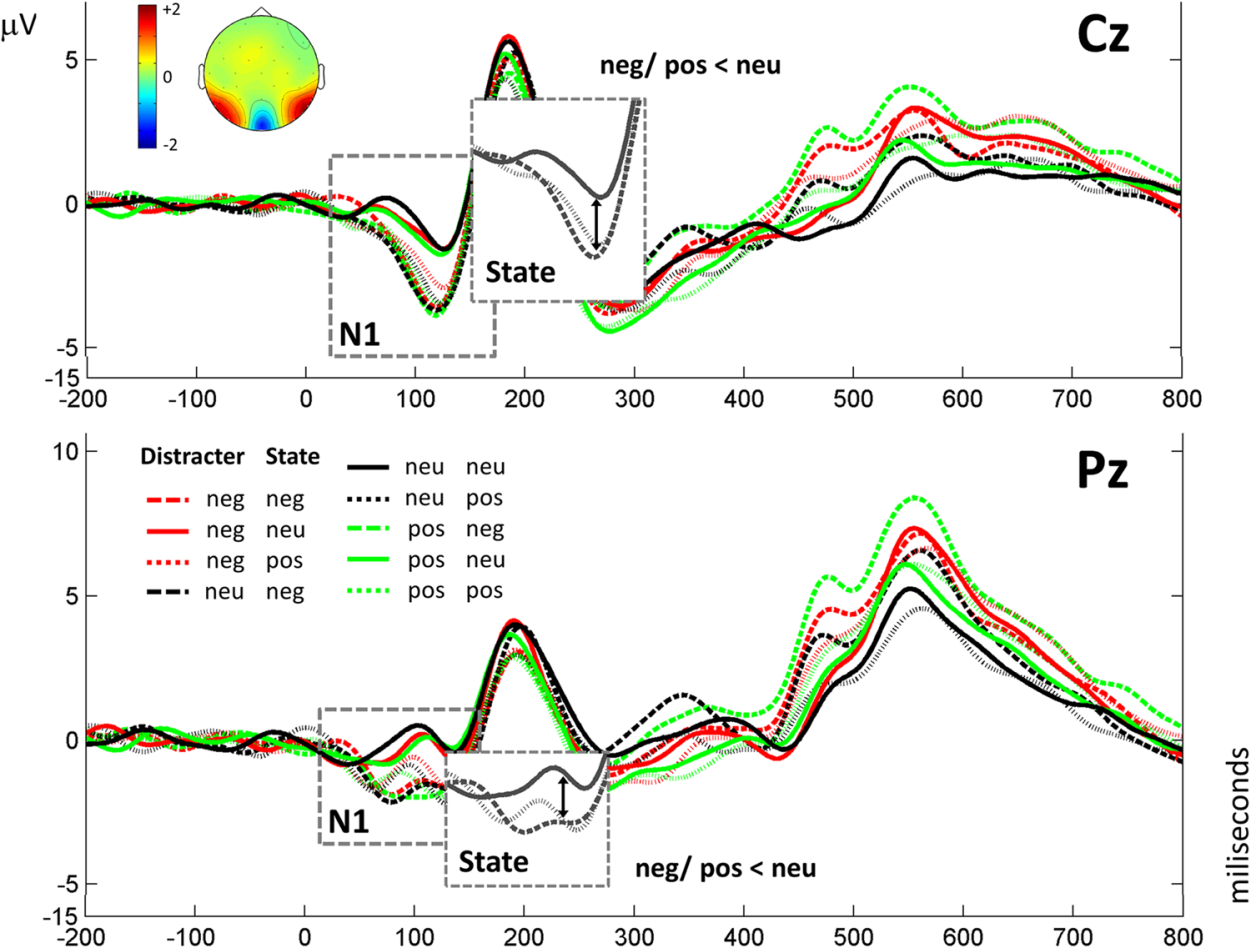
Results obtained for N1. Grand averages are presented at Cz and Pz, where results are clearly visible, depicting all conditions. Significant effects are shown in detail.

**Figure 2:**
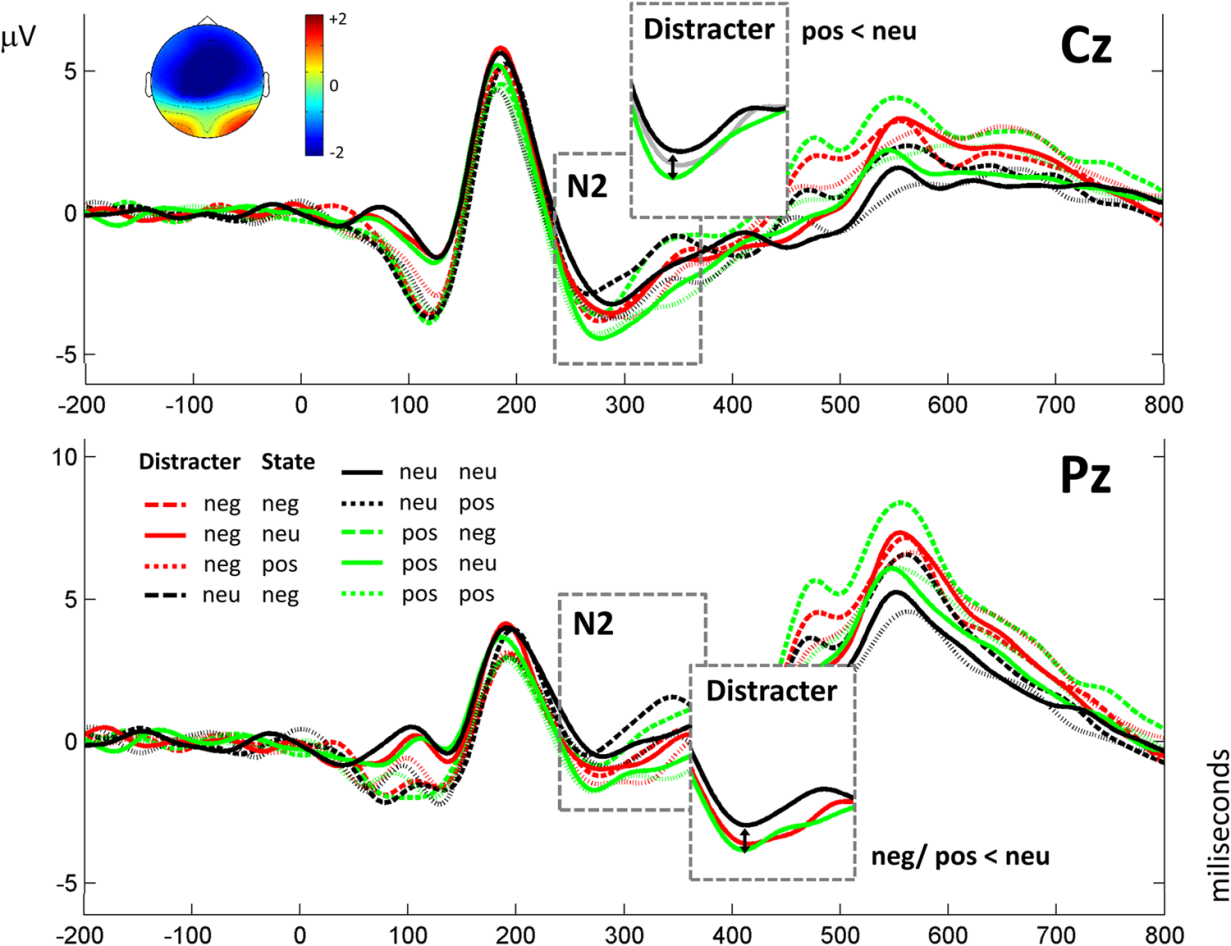
N2 results. Grand averages are presented at Cz and Pz, where results are clearly visible, including all conditions. Significant results at each topography are depicted in detail.

**Figure 3:**
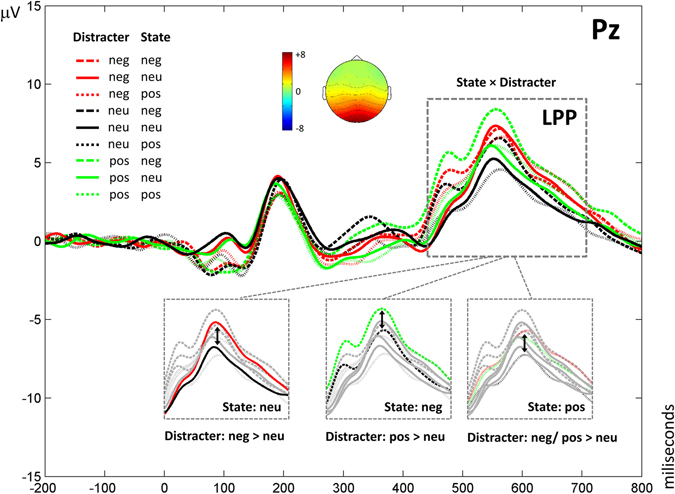
Results found in LPP. Grand averages are presented at Pz, where results are clearly visible. Significant interactions are shown in detail.

**Figure 4:**
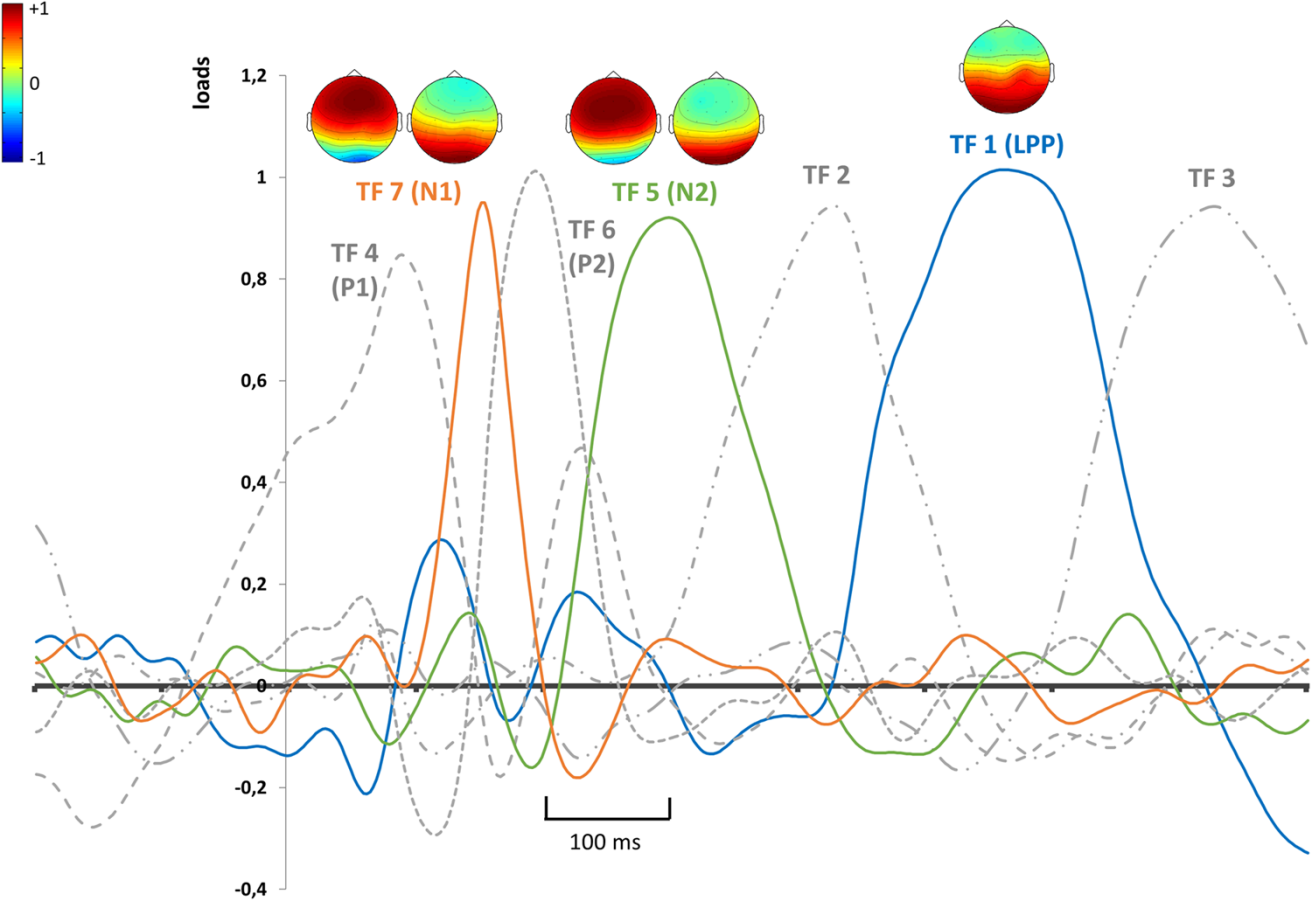
PCA factor loadings after promax rotation. Lines depict temporal loadings, and topographies represent spatial loadings. Temporal factors showing significant results are highlighted in color.

Figures 1–3 show grand averages of each ERP. These grand averages correspond to sites, where the experimental effects were more visible. As explained in the Methods section below, to detect and quantify outstanding ERP components, tPCA was applied to EEG signal. Based on the scree test, seven temporal factors (TFs) were extracted from the ERPs, explaining 85% of the total variance. The remaining components did not surpass the scree test cutoff because they did not account for a significant proportion of additional variance (i.e., the next one did not add more than 2% of additional variance). The sPCAs subsequently applied to all temporal factor scores extracted two spatial factors (SFs) for each of the temporal factors: one anterior or fronto-central and another posterior or parieto-occipital, explaining between 87 and 88% of variance. Temporal factor loadings of each component are represented as line plot in Fig. 4, and spatial factor loadings are shown as topographical plots. Original ERP amplitudes are a joint function of this factor loading waveform/factor loading topographies and the factor scores multiplied together. Factor peak latency and topography characteristics associated the following components with those contained in our hypothesis: TF4 (peaking at 90 ms) was associated with the P1 component, TF6 (192 ms) with P2, TF5 (294 ms) with N2, and TF1 (563 ms) with LPP. These labels will be employed hereafter to make results easier to understand. Importantly, around C1 latencies, no component was determined through tPCA.

#### Experimental effects on scalp ERP components

Spatial factor scores (linearly related to amplitudes) of each of these relevant temporal factors (i.e., P1, P2, N2, and LPP) were submitted to ANOVAs on State (negative, neutral, positive) × Distractor (negative, neutral, positive). Among the components relevant to our hypothesis, N2 and LPP were those showing significant results. Spatial factor scores of the remaining temporal factors were also submitted to ANOVAs, in order to reveal additional novel information contained in our data. In fact, results for TF7 (peaking at 154 ms and, therefore, associated with N1) were also significant. Table 1 presents means and standard deviations of significant components, and Figs 1–3 show a summary of all significant results.

Specifically, ANOVAs on N1 yielded a significant main effect of State at both anterior and posterior scalp regions, as illustrated in Fig. 1 [anterior N1: *F*(2,58) = 10.9, *p* < 0.001, *η* ^*2*^_*p*_ = 0.273; posterior N1: *F*(2,58) = 6.8, *p* < 0.005, *η* ^*2*^_*p*_ = 0.190]. Bonferroni corrected post-hoc tests indicated that N1 amplitudes were significantly greater when the participants’ state was emotional (negative and positive) than when it was neutral [anterior N1: *p* < 0.001 and *p* < 0.005, respectively; posterior N1: *p* < 0.05 and *p* < 0.005, respectively]. There were no significant differences between emotional and neutral Distractors, nor any significant interaction of State and Distractor [all *p* > 0.05].

Results regarding N2 indicated a significant main effect of Distractor at both anterior and posterior scalp regions [anterior N2: *F*(2,58) = 3.6, *p* < 0.05, *η* ^*2*^_*p*_ = 0.111; posterior N2: *F*(2,58) = 5.4, *p* < 0.01, *η* ^*2*^_*p*_ = 0.157]. As depicted in Fig. 2, and as evidenced by Bonferroni post hoc tests, positive distractors, rather than neutral ones, elicited more negative N2 amplitudes at fronto-central sites [*p* < 0.05], and negative and positive distractors, compared to neutral ones, at parieto-occipital sites [*p* < 0.05 in both cases].

Finally, a significant interaction effect of State × Distractor was found in parietal LPP (Fig. 3) [*F*(4,116) = 4.6, GG corrected *p* < 0.01, *η* ^*2*^_*p*_ = 0.136]. Specifically, when the participants’ state was negative, amplitudes in response to positive distractors were greater than to neutral ones, as shown by Bonferroni corrected post-hoc comparisons [*p* < 0.005]; when the state was neutral, negative distractors elicited greater amplitudes than neutral ones [*p* < 0.05]; and when experiencing a positive state, both negative and positive distractors elicited higher amplitudes than neutral ones [*p* < 0.001 and *p* < 0.005, respectively]. Comparing the effect of distractors on LPP between affective states, the interaction effect was confirmed: the difference of negative versus neutral distractors varied significantly from the negative to the positive mood [*F*(1,29) = 8.0, *p* < 0.01, *η* ^*2*^_*p*_ = 0.216], while the difference between positive and neutral stimuli did not vary [*p* > 0.05]; further, the difference between negative and positive compared to neutral distractors also varied significantly from the positive to the neutral state, being significant when distractors were positive (compared to neutral) [*F*(1,29) = 4.3, *p* < 0.05, *η* ^*2*^_*p*_ = 0.129], and non-significant when they were negative (compared to neutral) [*p* > 0.05].

### SCL data

Paired-samples *t*-tests computed on SCL data showed that SCLs were significantly higher when participants were exposed to the arousing (positive and negative) movie fragments (mean = 3.41 μS, SD = 2.80) compared to the neutral one (mean = 2.57 µS, SD = 2.58) [*t*(27) = 2.5, *p* < 0.05], and this difference was still observed when starting the experimental task of each affective block (arousing: mean = 5.23 µS, SD = 3.60; neutral: mean = 4.24 µS, SD = 3.45) [*t*(27) = 3.4, *p* < 0.01]. Further, one-way repeated measures ANOVAs revealed a significant effect of State during the experimental task [*F*(2,54) = 5.1, *p* < 0.01, *η* ^*2*^_*p*_ = 0.160]. Bonferroni-corrected post hoc tests indicated that SCL was significantly higher during the experimental task of the negative (mean = 5.08 µS, SD = 4.00) and the positive block (mean = 5.37 µS, SD = 3.47) than during the neutral block (mean = 4.24 µS, SD = 3.45) [both *p* < 0.05]. Finally, correlation analyses on behavioral data and SCL measures yielded a significant correlation between reaction times and SCL during the task [*r*(250) = 0.196, *p* < 0.005]. Moreover, a significant correlation was observed between the amplitude of outstanding ERP components (anterior and posterior N1 and posterior LPP) and SCL during the task [anterior N1: *r*(250) = 0.204, *p* < 0.005; posterior N1: *r*(250) = 0.140, *p* < 0.05; posterior LPP: *r*(250) = 0.300, *p* < 0.001]. Correlations were non-significant for error rates, anterior and posterior N2, and anterior LPP (*p* > 0.05).

## Discussion

The present study explored the temporal dynamics of exogenous attention to affective distractors during a digit categorization task, whereby participants experienced three different mood states (negative, positive, neutral) induced by movie scenes. SCL data indicated that affective states were successfully modulated. Overall, behavioral results revealed that exogenous attention was preferentially directed to negative distractors, and ERP data confirmed this modulation by emotion, as reflected in anterior and posterior N2. Importantly, at even earlier stages (anterior and posterior N1), ERP data indicated that cognitive processing was increased when experiencing an emotional state. Additionally, an interaction between the affective state and the attention process was evident at later stages (posterior LPP). These results are further discussed during the following section.

As regards the efficiency of affective state induction, results show that SCL was increased during the negative and during the positive block, compared to the neutral block. This is consistent with previous evidence obtained when employing this autonomic index for assessing emotional arousal (e.g., refs 62, 66). These results indicate that affective state was appropriately induced, though, the present design does not allow to determine if the described mood effects were generated by the movie clips presented at the beginning of each block or by the movie stills of the same valence interspersed between trial and trial.

With respect to exogenous attention effects, and at the behavioral level, reaction times and error rates showed that negative distractors, rather than positive and neutral ones, elicited the lowest accuracy levels and the longest reaction times in the digit categorization task, in line with the most common result obtained in previous studies employing similar CDTD tasks^6–15^. This pattern points to a negativity bias, a term that describes that unpleasant stimuli tend to elicit enhanced responses with respect to neutral or even positive ones^67, 68^, in this case reflected in enhanced attention to distractors. Furthermore, a significant positive correlation was observed between SCL measures during the task and reaction times; this linkage may imply that the enhanced arousal associated with the emotional states (negative and positive) is related to improved attention to emotional distractors (reflected in impaired behavioral performance).

At the neural level, a main effect of affective state on the early anterior and posterior N1 components (peaking approximately at 150 ms from stimulus onset) was found; thus, emotionally arousing moods (positive and negative) elicited greater N1 amplitudes than the neutral mood. This is in line with previous evidence showing that experiencing an emotional state may broaden early processing of stimuli^52, 69^, and specifically, of distracting stimuli^55, 57, 70^, though these previous studies described effects at even earlier latencies (C1 and P1). This discrepancy might be due to differences in the experimental paradigms (i.e., previous studies mentioned above did not present any kind of distractors or the employed distractors were not emotional). The present result suggests that the emotional basal state may modulate preattentional evaluation processes of visual stimuli, a modulation that, during the neural time course, was even prior to the display of exogenous attention. Moreover, the significant correlation, observed between N1 at both anterior and posterior sites and SCL during the task, corroborates this result. Accordingly, assuming that SCL is modulated by mood (e.g., refs 62–66), the simultaneous variation of SCL and N1 amplitudes provides additional evidence supporting this early modulation by the current mood state. This emotionally mediated amplification of very early processing resources during an affective state might be interpreted from an evolutionary point of view as an adaptive mechanism, which avoids being distracted by the mood state. However, some previous studies have reported a reduction of processing resources associated to negative mood (e.g., refs 44, 54). Differences between study outcomes might be related to the experimental paradigm, as already mentioned (i.e., the present study employed a CDTD task with emotional content), or to the arousal dimension of the affective state; it should be noted that in the present study the induced effect was highly arousing for both the negative and positive state, while in other studies it may have been less arousing. In the present study, in which the arousal levels of negative and positive moods were intentionally matched ‒as usual when introducing negative and positive valence at a time in the same experimental design‒, the effects of the valence and the arousal dimensions on N1 amplitude cannot be differentiated.

Added to this, the anterior and posterior N2 component (peaking approximately at 300 ms) reflected a modulating effect elicited by emotional distractors. This component is typically described as sensitive to exogenous attention to task-irrelevant emotional distractors in CDTD tasks, both at anterior (fronto-temporal) and posterior regions^6, 23, 26–29^, and has mostly been proposed to be involved in sensory amplification and reorienting towards the distractor^23, 27^. The participants’ emotional state did not influence the exogenous attention process associated with this component, which may also reflect an advantage from an evolutionary perspective; thus, the fact that emotional, potentially relevant distractors are attended independently of the current mood state at early attentional stages (when distractors have not been consciously processed yet) seems an adaptive and life-sustaining mechanism. It should be mentioned at this point that some previous studies employing other ‒non-CDTD‒ paradigms reported increased amplitudes at early latencies only in response to negative relative to neutral task-irrelevant stimuli^71^, while task-irrelevant positive compared to neutral stimuli resulted in a reduction of N1 and N2 in an identical paradigm^72^. Therefore, the advantage of positive stimulation must be understood as closely linked to experimental demands.

In summation, the late posterior LPP (peaking at 560 ms) showed a significant interaction effect of affective state and distractor; specifically, when participants experienced a negative mood state, the amplitude of the component in response to positive stimuli was greater than in response to neutral stimuli; during a positive state, both negative and positive pictures evoked higher amplitudes compared to neutral ones; and when the participants’ mood state was neutral, negative stimuli elicited greater amplitudes relative to neutral ones. Further, posterior LPP data correlated significantly with SCL arousal measures during the task, supporting the amplitude modulation by mood, as in the case of N1. LPP amplitudes have been previously reported to reflect the intensity of subjective emotional experience, its magnitude predicting the arousal of self-reported emotion evoked by relevant stimuli^73, 74^. In addition, in CDTD tasks, there is previous evidence relating posterior LPP amplitude to the processing of the distractor^7^. The present LPP results are therefore of special interest. As mentioned before, while there are numerous studies manifesting the existence of an interaction effect between attention and mood state at the endogenous level, at the exogenous level, evidence is very scarce and, to the best of our knowledge, no previous studies have described the temporal course of this interaction. Accordingly, the present findings can be considered as novel, and might have several implications. First, results found for the neutral mood state indicate that participants processed negative distractors more deeply than neutral ones; this is similar to the scarce previous evidence reported when employing a similar task but without modulating the affective state (i.e., when the mood state was assumed to be neutral as well; 7). Importantly, although endogenous attention to the distractor cannot be discarded at LPP latencies^5, 75^, the cited and the present results differ from those obtained when emotional stimuli were presented as endogenously attended targets (e.g., refs 35–42) or as task-irrelevant stimuli at fixation^26, 30–34^, where an advantage of both negative and positive stimuli, compared to neutral ones, was generally prominent. Moreover, this neural LPP result is consistent with the behavioral result, which points to exactly the same bias. Indeed, behavioral outcomes measured in attention-to-emotion tasks have been previously associated with ERP outcomes at LPP latencies, rather than at earlier latencies (e.g., ref. 76), given that reaction times and error rates occur with a delay of several hundred milliseconds compared to the actual first allocation of attention to a stimulus. Second, the negative state seems to be related to a suppression of the processing advantage of negative distractors, which was observed in the neutral mood condition, and, in turn, to a deeper evaluation of positive distractors than of neutral ones. This is in line with previous behavioral evidence showing that attention is automatically devoted to information, which is opposite in valence to the current motivational or mood state^51, 77, 78^. This counter-regulation might indicate an unconscious adaptive mechanism, which serves to compensate the negative mood and to regulate emotion and action, preventing them from escalating. An interesting further step would be to explore if this mechanism is altered in depression or anxiety disorders, in which individuals tend to exaggeratedly focus on negative information. Third, when the induced mood was of positive valence, participants were able to process negative and positive distractors to a greater extent than neutral ones; thus, it seems that, in this state, more resources were available to lead with emotional information. This is consistent with the existing evidence at the endogenous level indicating that positive mood states are able to broaden attention, to reduce the selectivity of attentional filters, and to increase the processing of distractor stimuli, relative to both negative and neutral moods (e.g., refs 44, 52, 79). This finding may also be understood as a processing advantage from an adaptive point of view. Finally, an interesting issue for future studies would be to investigate the influence of one affective state over the other (i.e., the n-1 block effect); for instance, experiencing a negative state after a neutral state might potentially differ from experiencing a negative state after a positive one.

In sum, results of the present study evidence that both the emotional content of exogenously attended distractors and the participants’ affective state modulate ERP amplitudes at early, automatic stages of processing, but in an independent manner. Interestingly, affective state influenced the ERP time course at even earlier stages than the modulation of automatic attentional resources by emotional distractors. In turn, at late latencies, emotion and affective state showed an interaction. Future research should further explore these findings, especially in relation to affective disorders.

## Methods

### Participants

Thirty-two students from the Universidad Autónoma de Madrid participated in this experiment, although data from only 30 were finally analyzed, as explained later. Ages of these 30 participants (19 women) ranged from 18 to 33 (mean = 24.8, SD = 3.6). All of them participated voluntarily after providing informed written consent and received course credit for their participation. They reported normal or corrected-to-normal visual acuity. All methods were performed in accordance with the Declaration of Helsinki^80^, containing the relevant guidelines and regulations to be taken into account in studies with human participants. Further, the study was previously approved by the Research Ethics Committee of the Universidad Autónoma de Madrid.

### Stimuli and Procedure

**Figure 5:**
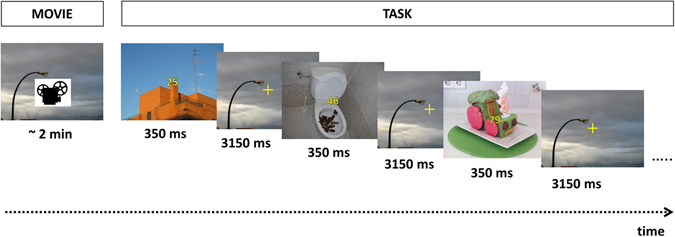
Schematic illustration of the task (this example shows the neutral block preceded by the neutral movie fragment). Note that these example pictures were not among the experimental stimuli.

Participants were placed in an electrically shielded, sound-attenuated and video-monitored room, 1 meter from a back-projection screen, where the task was presented through a RGB projector. It was designed and presented using Inquisit task programming software^81^. Figure 5 shows a schematic representation of the experimental design.

The experiment was composed of three blocks, and the emotional state of participants during the task was modulated through three emotionally charged movie fragments (negative, neutral, and positive), each of them presented at the beginning of one of the three blocks. The order of the resulting three affective blocks was counterbalanced across participants. Average duration of movie fragments was two minutes. Participants were asked to pay attention to the movies.

Immediately after the movie fragment corresponding to the current block, the task was presented. As shown in Fig. 5, each trial included two digits appearing in the center of the screen (relevant to the task) against a background of negative, neutral, or positive distractor images (irrelevant to the task). Thus, there were three different types of trials as a function of the distractor: negative, neutral, and positive. Size of distractors was of 38.1° × 29.1° visual degrees (width × height). The two central digits (2.3° height) were of yellow color and outlined in solid black, so that they could be clearly distinguished from the background. Each presentation (digits + distractor picture) was displayed on the screen during 350 ms, followed by a fixation cross of 3150 ms, so that the resulting stimulus onset asynchrony was 3500 ms. To keep the emotional state originated by the movie clip active during the corresponding block, the fixation cross was plotted against a single representative (in emotional terms) frame of the movie fragment shown at the beginning of the current block.

Participants were asked to press–as accurately and rapidly as possible–one key if both digits were even or if both were odd (i.e., if they were concordant), and to press another key if one digit was even and the other one was odd (i.e., if they were discordant). Previous evidence suggests that this kind of congruent-incongruent categorization requires conflict processing and control processes, which may modulate behavioral data (e.g., ref. 82). Further, the N2 component has repeatedly been reported to be highly sensitive to the processing of incongruence and conflict (e.g., refs 83–85), and this N2 effect has also been found to be modulated by task-irrelevant negative and positive emotions^86, 87^. Therefore, concordant and discordant digits were carefully matched between emotional distractor conditions and mood blocks, in order to discard the potential influence of incongruence on behavioral and N2 results. There were a total of 40 combinations of digits, half of them concordant and the other half discordant. The two digits were always different, and the same combinations of digits were repeated across all emotional conditions (negative, neutral, and positive distractors) to ensure that task demands were matched. Accordingly, there were 40 trials of each type consisting of 20 different pictures, each of them presented twice: with concordant and discordant digits. In each affective block, the same total number of 120 trials was displayed in two runs, separated by a short rest period (of seconds) with the purpose of ensuring that the participant was comfortable. Statistical control analyses computed on behavioral data confirmed that, as expected based on previous data^82^, reaction times and error rates were higher for discordant than for concordant digits [*F*(1,29) = 34.3, *p* < 0.001, *η* ^2^_p_ = 0.542; and *F*(1,29) = 5.9, *p* < 0.05, *η* ^2^_p_ = 0.169, respectively], but there were no differences between distractor conditions and mood states (i.e., the concordant-discordant difference was the same for all distractor × mood levels) [*p* > 0.05, for both reaction times and error rates, respectively]. Stimuli were presented in semi-random order in such a way that there were never more than two consecutive trials of the same emotional or numerical category. In sum, experimental conditions were nine: State (negative, neutral, positive) × Distractor (negative, neutral, positive). Participants were also instructed to look at the fixation cross located at the center of the screen and to blink preferably after a beep that sounded 1500 ms after each stimulus onset, in order to minimize ocular interference. Before starting the experiment, they completed a practice block using neutral distractors (different to those employed during the experimental phase), in order to ensure they understood the task instructions.

Background distractor pictures were selected from EmoMadrid database (www.uam.es/CEACO/EmoMadrid.htm) according to valence (ranging from negative to positive) and arousal (ranging from relaxing to arousing) average normative ratings (for database codes of pictures please see supplementary information). These two theoretically orthogonal affective dimensions are widely used to explain the principal variance of emotional meaning^88–91^. Importantly, at the end of the recording session, participants themselves filled out a bidimensional scale for each image, providing their own assessments on valence and arousal (Table 2). Statistical analyses were carried out on these ratings to confirm, first, that stimulus valence was as assumed a priori, and second, that positive and negative pictures were balanced with respect to their arousal levels. One-way repeated-measures ANOVAs were computed for valence and arousal dimensions, using Emotion (negative, neutral, positive) as factor. ANOVAs yielded significant differences in both valence and arousal [*F*(2,58) = 457.3, GG corrected *p* < 0.001, *η* ^*2*^_*p*_ = 0.940; and *F*(2,58) = 134.9, GG corrected *p* < 0.001, *η* ^*2*^_*p*_ = 0.823, respectively]. As expected, Bonferroni corrected post-hoc contrasts indicated that negative and positive pictures showed different valence [*p* < 0.001] but not different arousal levels [*p* > 0.05], and that they differed from neutral pictures in both dimensions [all *p* < 0.001].

**Table 2:** Means and standard deviations (in parenthesis) of subjective ratings of movie clips and pictures employed as distracters.

	Negative		Neutral		Positive	
**Subjective ratings of movies**						
Valence (1=negative to 5=positive)	1.43	(0.69)	3.43	(0.73)	4.57	(0.86)
Arousal (1=calming to 5= arousing)	4.63	(0.49)	3.20	(0.76)	4.30	(1.09)
**Subjective ratings of distracter pictures**						
Valence (1=negative to 5=positive)	1.80	(0.36)	3.18	(0.21)	4.07	(0.35)
Arousal (1=calming to 5= arousing)	4.19	(0.34)	2.92	(0.18)	4.00	(0.45)

Movie fragments were selected from a wider sample of 128 fragments previously assessed by 200 students from the Universidad Autónoma de Madrid (please see supplementary information for details about movie clips). As in the case of pictures, at the end of the experiment, participants also filled out a bidimensional scale for each fragment, providing their own assessments on valence and arousal (Table 2). Results of the one-way repeated-measures ANOVAs on these ratings confirmed the expected differences [valence: *F*(2,58) = 120.6, GG corrected *p* < 0.001, *η* ^*2*^_*p*_ = 0.806; arousal *F*(2,58) = 20.7, *p* < 0.001, *η* ^*2*^_*p*_ = 0.506], ratings of the negative and the positive fragment being different in valence [*p* < 0.001] but not in arousal [*p* > 0.05], and different from neutral ratings in both dimensions [all *p* < 0.001].

### ERP recording and pre-processing

Electroencephalographic (EEG) activity was recorded using an electrode cap (ElectroCap International) with tin electrodes. Thirty electrodes were placed at the scalp following an homogeneous distribution based on the International 10–20 System. All scalp electrodes were referenced to the nose tip. Electrooculographic (EOG) data were recorded supra- and infraorbitally (vertical EOG) as well as from the left versus right orbital rim (horizontal EOG). An online analog bandpass filter of 0.3 to 40 Hz was applied. Recordings were continuously digitized at a sampling rate of 420 Hz. The continuous recording was divided into 1000 ms epochs for each trial, beginning 200 ms before stimulus onset. A baseline correction was performed based on the prestimulus recording. Behavioral activity was recorded through a two-button keypad. Outlier trials (responses before 250 ms or after 2000 ms), erroneous trials, and trials with no response were excluded from the analyses. An offline digital bandpass filter of 0.3 to 30 Hz was applied using Fieldtrip software (http://fieldtrip.fcdonders.nl ^92^).

Ocular artifact removal was conducted through an Independent Component Analysis based strategy^93^ as implemented in Fieldtrip. After this process, a second stage of visual inspection of EEG data was conducted. If any further artifact was present, the corresponding trial was discarded. The average number of trials accepted within each stimulus category after this rejection of artifacts and incorrect responses is included in Table 1. A minimum criterion of 20 correct and artifact-free trials per condition and participant was set to ensure a reasonable signal-to-noise ratio of the ERP averages. Data from two participants could not be analyzed for this reason.

### SCL recording

SCL was recorded through tin electrodes attached to the palmar surface of the middle phalanges of the second and third fingers of the non-dominant hand, and continuously digitized at a sampling rate of 10 Hz. Data from two participants were lost due to technical failures (please note that SCL was not the variable of interest, but a mere control measure for the variable State).

### Data analysis

In all statistical analyses, post hoc comparisons were performed to determine the significance of pairwise contrasts using the Bonferroni correction procedure. In order to break down interaction terms, simple effects analyses were conducted (i.e., comparisons of the effects of one independent variable between the levels of the other). Effect sizes were computed using the partial eta-square (*η* ^*2*^_*p*_) method. The analyses were carried out using SPSS 19.0 software package^94^.

### Behavioral data

Reaction times and error rates were submitted to two-way repeated 3 × 3 ANOVAs introducing State (negative, neutral, positive) and Distractor (negative, neutral, positive) as factors. Given that behavioral data lacked normal distribution, data transformations were applied to the original values in order to achieve normality. Reaction times (RT; ms) were logarithmically transformed (log10[RT]), as recommended for this kind of distribution^95^, and error rates (ER; ranging from 0 to 1) were arcsin-root transformed (arcsin[√ER]), as appropriate for data, which lie between an upper and lower bound^96^.

### ERP data

#### Detection, spatio-temporal characterization, and quantification of relevant ERP components

In order to detect and quantify relevant ERP components, those explaining most of the variance in the temporal and spatial domain were extracted through covariance-matrix-based Principal Components Analysis (PCA). This technique has repeatedly been recommended for these purposes (e.g., refs 97–102). PCA determines components mathematically, avoiding subjectivity or inter-judge discrepancies often characterizing the traditional window/ region definition based on manual or visual criteria. In the first step, temporal PCA (tPCA) computes the covariance between ERP time points, which tends to be high between those involved in the same component and low between those belonging to different components. The solution is therefore a set of nearly independent factors made up of highly covarying time points, which ideally correspond to ERP components. Extracted temporal factors are quantified in factor loadings and factor scores, which are linearly related to amplitudes: amplitudes are a joint function of factor loadings and factor scores multiplied together (e.g., refs 101–103). The decision on the number of factors to select was based on the scree test^104^. Extracted factors were submitted to promax rotation^99–102^.

Once quantified in temporal terms, and prior to statistical contrasts on experimental effects, temporal factor scores were submitted to spatial PCA (sPCA), in order to decompose topographies at the scalp level into its main spatial regions. Thus, while tPCA separates ERP components along time, sPCA reliably separates them along space, each region or spatial factor ideally reflecting one of the concurrent neural processes underlying each temporal factor. This spatial decomposition is an advisable strategy prior to statistical contrasts, since ERP components frequently behave differently in some scalp areas than in others (e.g., they present opposite polarity or react heterogeneously to experimental manipulations). Basically, each spatial factor is formed by the scalp points, where recordings tend to covary; thus, the shape of the sPCA-configured regions is functionally based. Moreover, each spatial factor can also be quantified through the spatial factor score, a single parameter that reflects the amplitude of the whole spatial factor. Similarly, the decision on the number of factors to select was based on the scree test, and extracted factors were submitted to promax rotation as well. Statistical analyses were computed on factor scores, which are linearly related to amplitudes, as explained above.

#### Experimental effects

Finally, two-way repeated 3 × 3 ANOVAs on spatial factor scores were carried out for each temporal factor with respect to State (negative, neutral, positive) and Distractor (negative, neutral, positive), accounting for multiple comparisons employing the Bonferroni correction procedure.

### SCL data

SCL measures were analyzed for each affective block during two 60 s periods: (i) starting from 30 s after the beginning of each movie clip, and (ii) at the beginning of the experimental task of each block. Mean SCL values were computed for these two 60-s-epochs with respect to a baseline of 30 s recorded at the beginning of the experimental session. These SCL data were then submitted to paired-samples *t*-tests with respect to Arousal (high arousing = positive + negative, low arousing = neutral), and to one-way repeated measures ANOVAs introducing State (negative, neutral, positive) as factor. Moreover, in order to test the linkage between SCL measures and behavioral data/critical ERP components, correlation analyses were carried out using Pearson’s correlation coefficient.

### Data availability

All data are available upon request and may be published online after the manuscript is accepted.

## Electronic supplementary material

Supplementary info
