# Supplementary material for: The influence of affective state on exogenous attention to emotional distractors: behavioral and electrophysiological correlates
Source: Sci Rep. 2017 Aug 14;7:8068. doi: 10.1038/s41598-017-07249-x (PMC5556118; doi:10.1038/s41598-017-07249-x)
Supplement: Supplementary file 1 — Supplementary info [file 41598_2017_7249_MOESM1_ESM.doc]

**The influence of affective state on exogenous attention to emotional distractors: behavioral and electrophysiological correlates**

Alejandra Carboni^a^, Dominique Kessel^a,b^, Almudena Capilla^b^, & Luis Carretié^b^

^a^ Universidad de la República del Uruguay, Montevideo, Uruguay

^b^ Universidad Autónoma de Madrid, Madrid, Spain

*Corresponding author

Alejandra Carboni

Centro de Investigación Básica en Psicología

Universidad de la República

11200 Montevideo (Uruguay)

alejandra.carboni@psico.edu.uy

**SUPPLEMENTARY INFORMATION**

**EmoMadrid database codes of pictures employed during the task:**

Negative: EM0017, EM0031, EM0090, EM0287, EM0318, EM0319, EM0357, EM0363, EM0391, EM0394, EM0395, EM0396, EM0432, EM0447, EM0484, EM0487, EM0562, EM0568, EM0579, EM0581.

Neutral: EM0035, EM0044, EM0053, EM0054, EM0076, EM0083, EM0085, EM0131, EM0137, EM0158, EM0192, EM0198, EM0209, EM0248, EM0285, EM0338, EM0365, EM0489, EM0550, EM0561.

Positive: EM0063, EM0091, EM0163, EM0234, EM0238, EM0240, EM0269, EM0279, EM0302, EM0353, EM0368, EM0384, EM0397, EM0399, EM0402, EM0413, EM0458, EM0460, EM0461, EM0462.

**Movie fragments employed during the task:**

Negative: Fight Club, 1999 (min 1:02:06-1:03:38)

Neutral: Catch me if you can, 2002 (min 0:56:09 –0:58:30)

Positive: Fame, 2009 (min 0:21:46– 0:24:10)
